# Chronic kidney outcomes associated with GLP-1 receptor agonists versus long-acting insulins among type 2 diabetes patients requiring intensive glycemic control: a nationwide cohort study

**DOI:** 10.1186/s12933-023-01991-5

**Published:** 2023-10-04

**Authors:** Zi-Yang Peng, Chun-Ting Yang, Wei-Hung Lin, Wen-Yu Yao, Huang-Tz Ou, Shihchen Kuo

**Affiliations:** 1 Institute of Clinical Pharmacy and Pharmaceutical Sciences, College of Medicine, National Cheng Kung University, 1 University Road, Tainan, 701 Taiwan; 2 Division of Nephrology, Department of Internal Medicine, National Cheng Kung University Hospital, College of Medicine, National Cheng Kung University, Tainan, Taiwan; 3 Institute of Clinical Medicine, College of Medicine, National Cheng Kung University, Tainan, Taiwan; 4 Department of Pharmacy, College of Medicine, National Cheng Kung University, Tainan, Taiwan; 5 Division of Metabolism, Endocrinology and Diabetes, Department of Internal Medicine, University of Michigan Medical School, Ann Arbor, MI USA

**Keywords:** Chronic kidney outcomes, Intensive glycemic control, GLP-1 receptor agonists, Long-acting insulins

## Abstract

**Background:**

Effectiveness of glucagon-like peptide-1 receptor agonists (GLP-1RAs) versus long-acting insulins (LAIs) on preventing progressive chronic kidney outcomes is uncertain for type 2 diabetes (T2D) patients requiring intensive glycemic control. This study aimed to evaluate comparative effectiveness of GLP-1RA versus LAI therapies on progressive chronic kidney outcomes among patients having poor glycemic control and requiring these injectable glucose-lowering agents (GLAs).

**Methods:**

7279 propensity-score-matched pairs of newly stable GLP-1RA and LAI users in 2013–2018 were identified from Taiwan’s National Health Insurance Research Database and followed until death or 12/31/2019 (intention-to-treat). Subdistributional hazard model was utilized to assess the comparative effectiveness on a composite renal outcome (i.e., renal insufficiency [eGFR < 15 mL/min/1.73 m^2^], dialysis-dependent end-stage renal disease [ESRD], or renal death) and its individual components. Sensitivity analyses with the as-treated scenario, PS weighting, high-dimensional PS techniques, using cardiovascular diseases (CVDs) as positive control outcomes, and interaction testing were performed.

**Results:**

In primary analyses, subdistribution hazard ratios (95% CIs) for initiating GLP-1RAs versus LAIs for the composite renal outcome, renal insufficiency, dialysis-dependent ESRD, and renal death were 0.39 (0.30–0.51), 0.43 (0.32–0.57), 0.29 (0.20–0.43), and 0.28 (0.15–0.51), respectively. Sensitivity analysis results were consistent with the primary findings. CVD history and the medication possession ratio of prior oral GLAs possessed modification effects on GLP-1RA-associated kidney outcomes.

**Conclusion:**

Using GLP-1RAs versus LAIs was associated with kidney benefits in T2D patients requiring intensive glycemic control and potentially at high risk of kidney progression. GLP-1RAs should be prioritized to patients with CVDs or adherence to prior oral GLAs to maximize kidney benefits.

**Supplementary Information:**

The online version contains supplementary material available at 10.1186/s12933-023-01991-5.

## Background

Persistent hyperglycemia despite the use of multiple glucose-lowering agents (GLAs) among patients with type 2 diabetes (T2D) could deteriorate renal functions and thereby increase the risk of renal diseases [1]. Hence, optimizing GLA therapies that can normalize glucose levels and improve glycemic control is essential for delaying the progression to renal failure in this population [2]. For such patients, long-acting insulins (LAIs) and glucagon-like peptide-1 receptor agonists (GLP-1RAs) are recommended as injectable GLAs to intensify glycemic control [3]. In particular, the use of GLP-1RAs and LAIs in Taiwan’s clinical practice setting is generally limited to T2D patients in the late treatment course (i.e., having persistent hyperglycemia under three types of oral GLAs or at the level of HbA1c ≥ 8.5% under oral GLA treatments [4]) requiring intensive glycemic control.

Possible renal benefits with the use of GLP-1RAs have been recognized in recent studies. Direct interactions of GLP-1RAs with human glomerulus and renal tubules have been shown to slow the progression to renal failure [5–7]. A decreased incidence of macroalbumiuria among GLP-1RA users compared to those with placebo was reported in both LEADER [8, 9] and REWIND trials [10]. Unfortunately, the generalizability of trial findings [8–10] to real-world settings, which comprise diverse patients and multiple competing treatments, is unclear. In addition, it is unclear whether short-term improvements in renal outcomes (e.g., macroalbuminuria) associated with GLP-1RA use obtained from limited follow-up trial periods can translate into the long-term benefits for clinically meaningful renal events (e.g., dialysis or renal death) in daily practice settings. Few real-world studies have assessed the chronic renal outcomes of GLP-1RA therapies versus other GLA therapies among general T2D populations [11–13], and there is a lack of data on the comparative renal effects of GLP-1RAs versus LAIs in patients who require intensive glycemic control and are at a high risk of the progression of renal diseases.

Against this background, we sought to generate real-world data on the comparative effectiveness of GLP-1RA versus LAI therapies on progressive chronic renal outcomes among patients with need for intensified injectable GLAs. In addition, exploratory analyses were performed to investigate the heterogeneity of treatment effects among real-world patient populations with diverse clinical characteristics to identify potential effect modifiers. Such information can facilitate clinical treatment decision-makings to enhance personalized medicine for patients.

## Methods

### Data source

This study was approved by the Institutional Review Board of National Cheng Kung University Hospital (B-ER-108-474). Taiwan’s National Health Insurance Research Database (NHIRD) was utilized in this study. Briefly, Taiwan’s NHIRD is a nationwide, population-based claims database that comprises de-identified, individual-level, and longitudinal health records (i.e., outpatient, emergency, and inpatient departments, and pharmacy refills) of each beneficiary enrolled in the National Health Insurance (NHI) program, which covers over 99% of the population in Taiwan [14]. Details of the NHIRD are available elsewhere [14].

### Cohort identification and follow-up

This comparative effectiveness research applied an active-comparator, new-user design [15]. Stable users of the study drugs (i.e., individuals with at least three prescriptions of GLP-1RAs or LAIs with any gaps between two consecutive drug refills of less than 30 days) were first identified from patients with T2D in the NHIRD in the period of 2013 to 2018. The initiation of the study drugs was defined as the index date. Patients younger than 18 years of age at the index date and those with undefined sex were excluded. Per the new-user design in this study, prevalent users of GLP-1RAs or LAIs in the year before the index date were excluded. Patients prescribed with both GLP-1RAs and LAIs at the index date were also excluded to avoid the misclassification of treatment exposure. We also excluded patients with concurrent use of a basal-bolus insulin regimen or premixed insulin at the index date because they were likely to have poor glycemic control or fluctuating blood glucose levels during mealtimes [3]. Next, to avoid prevalent cases with renal insufficiency in the present study, study subjects with one of the following characteristics at baseline were excluded: prior use of erythropoiesis stimulating agents (ESAs), end-stage renal disease (ESRD), or renal transplantation in the year before the index date. Moreover, to enhance the comparability of study cohorts, 5-to-1-digit greedy propensity score matching (PSM) procedures were implemented to obtain GLP-1RA- and LAI-matched pairs at a 1:1 ratio [16]. Briefly, each patient’s PS, which indicated the probability of receiving the treatment of interest (i.e., GLP-1RAs versus LAIs), was estimated using a logistic regression analysis, where a series of patient characteristics at baseline (i.e., demographics at the index date, diabetes-related complications, exposure to GLAs, medication possession ratio [MPR] of prior GLA use, which was calculated as the day supply of GLAs divided by 365 days [17], and exposure to cardiovascular [CV] and kidney-disease-associated medications in the year prior to the index date) were treated as explanatory variables for treatment status (i.e., GLP-1RAs or LAIs). Among these variables, the number of oral GLA prescriptions in the year before the index date and the status of prandial insulin use in the month before the index date were specified as proxies for the diabetes severity of individual patients, as recommended by the literature [4] and clinical experts. The details of cohort selection are available in Additional file 1: Fig. S1. Each patient was followed from the initiation of the study drugs until the occurrence of study outcomes, loss of follow-up, death, or the end of 2019, whichever came first (i.e., intention-to-treat [ITT] scenario).

### Drug exposure and study outcome assessment

Drug exposure was measured according to the World Health Organization Anatomical Therapeutic Chemical Classification System. Of noted, only human-based GLP-1RAs (i.e., liraglutide and dulaglutide) available during study period in Taiwan were included in analysis. Study outcomes of interest were the composite progressive chronic renal outcome, including (1) renal insufficiency which referred to estimated glomerular filtration rate (eGFR) < 15 mL/min/1.73 m^2^ and was determined by the stable use of ESAs (i.e., at least two prescriptions of darbepoetin alfa or methoxy polyethylene glycol-epoetin beta, or four prescriptions of erythropoietin within 3 months) [18], given that the reimbursement policy of Taiwan’s NHI program restricts the use of ESAs only to patients with stage 5 chronic kidney disease (CKD), (2) dialysis-dependent ESRD, which was ascertained using the Registry of Catastrophic Illness Patients, and (3) renal death, which was determined according to the Cause of Death in the NHIRD, and individual components of the composite outcome. The operational definitions of the study outcomes are detailed in Additional file 1: Table S1.

### Statistical analyses

The standardized mean difference (SMD) was employed to examine the between-group difference in baseline patient characteristics before and after the PSM, with an absolute SMD value greater than 0.1 indicating a statistically significant between-group difference. The event rates of study outcomes in each treatment group were calculated as the number of events per 100 person-years. Given the high mortality risk of our study subjects, who were likely to be severe cases requiring intensive glycemic control with injectable GLAs (i.e., GLP-1RAs or LAIs) in the late treatment course of diabetes [19], the primary analyses under the ITT scenario employed subdistribution hazard models instead of traditional Cox models [20], to account for the possibility of competing risk of death to study events in our study cohort. The results are presented as subdistribution hazard ratios (SDHRs) and associated 95% CIs.

A series of sensitivity analyses were performed to test the robustness of the study findings. First, an as-treated (AST) scenario analysis was performed, where each patient was followed from the index date until the discontinuation of the study drug, a switch to or addition of another study drug, the occurrence of study outcomes, loss of follow-up, death, or the end of 2019, whichever came first. Second, to retain the most GLP-1RA and LAI users during our study period and thereby all observed events in the analysis, three PS weighting approaches, namely inverse probability of treatment weighting (IPTW), stabilized IPTW, and standardized mortality ratio weighting (SMRW) [21], were utilized to obtain the SDHRs of the study outcomes. Third, to minimize the possibility of unmeasured confounding effects that may arise with the use of the NHIRD, we estimated the high-dimensional PS (hdPS) using baseline patient characteristics that were associated with the receipt of the treatment of interest (i.e., GLP-1RAs versus LAIs) and simultaneously generated from empirical variables in five data dimensions of the NHIRD (i.e., outpatient diagnoses, outpatient procedures, inpatient diagnoses, inpatient procedures, and medication use) [22]. We re-matched the study cohorts using hdPS and estimated the SDHR accordingly. Fourth, to investigate the heterogeneity of GLP-1RA-associated renal effects by baseline patient characteristics, the primary analyses were stratified by patients’ age, sex, history of diabetes-related complications, and MPR of previously used GLAs (MPR ≥ 0.8 versus < 0.8 [17]) and use of renin-angiotensin aldosterone system agents, where these characteristics were modeled with treatment status (i.e., GLP-1RAs versus LAIs) as interaction terms in the analysis. These characteristics have been recognized as potential effect modifiers for GLP-1RA-associated outcomes according to existing evidence [11, 23] and are recommended by clinical experts. Lastly, analyses of clinical outcomes that are well known to be positively associated with GLP1-RA therapy (i.e., positive control outcomes) were conducted to examine the validity of the study procedures [24]. That is, given the apparent GLP-1RA-associated CV benefits [4, 25], the three-point major adverse CV event (3P-MACE, including nonfatal myocardial infarction [MI], nonfatal stroke, and CV death) and individual components of 3P-MACE were selected as positive control outcomes for analysis. All of the above-mentioned analyses were performed using SAS software version 9.4.

## Results

**Fig. 1:**
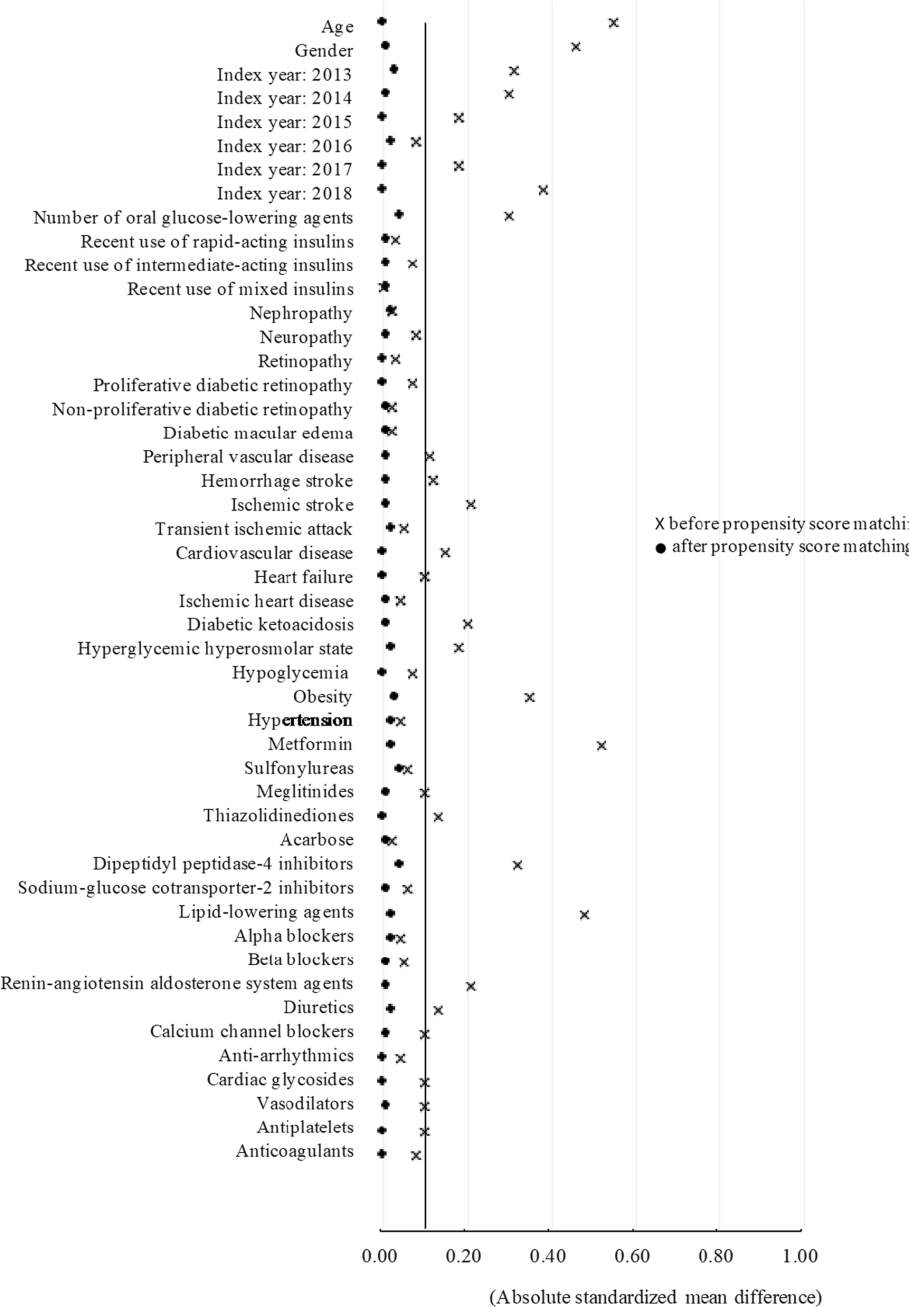
Absolute standardized mean differences of baseline characteristics between GLP-RA and LAI groups before and after propensity score matching. Black vertical line indicates standardized mean difference of 0.1. Values (i.e., black dots or crosses) greater than 0.1 indicate statistically significant between-group difference in patient baseline characteristics

There were 99,889 of T2D patients with stable use of GLP-1RAs or LAIs identified from the NHIRD in 2013 ~ 2018. After applying study exclusion criteria (e.g., the use of basal-bolus insulin regimens or premixed insulins at index date [i.e., 12,593 subjects] shown in Additional file 1: Fig. S1), 7643 and 49,570 of incident new-users of GLP-1RAs and LAIs, respectively, were obtained for further PSM. A total of 7279 PS-matched pairs of GLP-1RA and LAI users were identified (Additional file 1: Fig. S1), and they had a satisfactory between-group comparability at baseline supported by all absolute SMD statistics less than 0.1 (Fig. 1). Kernel density plots of the PS distribution before and after the matching are shown in Additional file 1: Fig. S2, and the proportions of individual GLP-1RAs and LAIs are detailed in Additional file 1: Table S2. As shown in Additional file 1: Table S3, study subjects after PSM had a mean age of 49 years, and 49% of them were female, and 19%, 25.6%, 8.1% and 11.3% of the subjects had a history of cardiovascular diseases (CVDs), nephropathy, neuropathy, and retinopathy, respectively. On average, each patient was prescribed nearly three types of GLA in the year before the index date.

### Primary and sensitivity analyses of chronic renal outcomes and positive control outcome analyses

The results of primary analyses with the ITT scenario show that over a mean follow-up period of 2.64 years, the SDHRs (95% CIs) of composite chronic renal outcome, renal insufficiency (i.e., eGFR < 15 mL/min/1.73 m^2^), dialysis-dependent ESRD, and renal death for GLP-1RA versus LAI therapies were 0.39 (0.30–0.51), 0.43 (0.32–0.57), 0.29 (0.20–0.43), and 0.28 (0.15–0.51), respectively (Table 1). Similarly, the SDHRs (95% CIs) of these outcomes obtained from the AST scenario analyses were 0.36 (0.25–0.53), 0.40 (0.27–0.60), 0.20 (0.11–0.38), and 0.47 (0.11–1.68), respectively (Additional file 1: Table S4).

**Table 1:** Event rates and hazard ratios of renal outcomes associated with use of GLP-1RAs versus LAIs (primary analyses with intention-to-treat scenario)

	GLP-1RAs (n = 7279)		LAIs (n = 7279)		SDHR (95% CI) of GLP-1RAs versus LAIs
	Number of events	Event rate (events/100 pys)	Number of events	Event rate (events/100 pys)	
Composite renal outcome^*^	79	0.41	199	1.06	0.39 (0.30–0.51)
Renal insufficiency (i.e., eGFR < 15 mL/min/1.73 m^2^)^†^	68	0.36	157	0.84	0.43 (0.32–0.57)
Dialysis-dependent ESRD	35	0.18	119	0.63	0.29 (0.20–0.43)
Renal death	13	0.07	47	0.25	0.28 (0.15–0.51)

*GLP-1RAs* glucagon-like peptide-1 receptor agonists, *LAIs* long-acting insulins, *SDHR* subdistribution hazard ratio, *pys* person-years, *eGFR* estimated glomerular filtration rate, *ESRD* end-stage renal disease

^*^Composite renal outcome includes renal insufficiency, dialysis-dependent ESRD, and renal death

^†^Renal insufficiency referred to eGFR < 15 mL/min/1.73 m^2^ and was determined by the stable use of erythropoiesis stimulating agents (ESAs) (i.e., at least two prescriptions of darbepoetin alfa or methoxy polyethylene glycol-epoetin beta, or four prescriptions of erythropoietin within three months), given that the reimbursement policy of Taiwan’s National Health Insurance program restricts the use of ESAs only to patients with stage 5 chronic kidney disease. This operational definition was also confirmed with clinical nephrologists

**Fig. 2:**
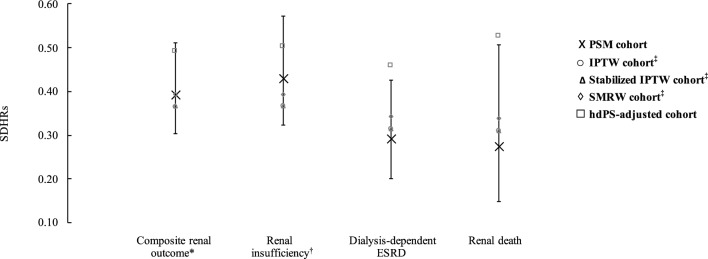
Results of subdistribution hazard model analyses for GLP-1RA versus LAI therapies on study outcomes (primary and sensitivity analyses). Black vertical dotted lines are 95% CIs of study outcomes obtained from primary analyses in propensity-score-matched cohorts. *SDHRs* subdistribution hazard ratios, *PSM* propensity score matching, *IPTW* inverse probability of treatment weighting, *SMRW* Standardized mortality ratio weighting, *hdPS* high-dimensional propensity score, *ESRD* end-stage renal disease. ^*^Composite renal outcome includes stable use of ESA, dialysis-dependent ESRD, and renal death. ^†^Renal insufficiency referred to estimated glomerular filtration rate < 15 mL/min/1.73 m^2^ and was determined by the stable use of erythropoiesis stimulating agents (ESAs) (i.e., at least two prescriptions of darbepoetin alfa or methoxy polyethylene glycol-epoetin beta, or four prescriptions of erythropoietin within three months), given that the reimbursement policy of Taiwan’s National Health Insurance program restricts the use of ESAs only to patients with stage 5 chronic kidney disease. This operational definition was also confirmed with clinical nephrologists. ^‡^We removed patients whose propensity scores were either more than 0.95 or less than 0.05; the weights were then estimated based on the trimmed populations. Weights in the IPTW approach were estimated as follows: Weight_GLP1RAs_ = 1/PS and Weight_LAIs_ = 1/(1-PS). Weights in the stabilized IPTW approach were estimated as follows: Weight_GLP1RAs_ = Prevalence of GLP1RA users (%)/PS and Weight_LAIs_ = Prevalence of LAI users (%)/(1-PS). Weights in the SMRW approach were estimated as follows: Weight_GLP1RAs_ = 1 and Weight_LAIs_ = PS/(1-PS)

Sensitivity analyses based on PS-weighted cohorts (using IPTW, stabilized ITPW, and SMRW approaches) and hdPS-matched cohorts give results that are consistent with the primary findings (Fig. 2). That is, the SDHR estimates of these four sensitivity analyses fell within the 95% CIs of the estimates from primary analyses using the PSM approach for all study outcomes including composite chronic renal outcome, renal insufficiency, dialysis-dependent ESRD, and renal death. SDHRs and associated 95% CIs in these sensitivity analyses are detailed in Additional file 1: Table S5.

Positive control outcome analyses show GLP-1RA-therapy-associated CV benefits; i.e., the SDHRs (95% CIs) of 3P-MACE, non-fatal MI, non-fatal stroke, and CV death for GLP-1RAs versus LAIs were 0.71 (0.60–0.84), 0.73 (0.54–0.99), 0.76 (0.60–0.95), and 0.49 (0.34–0.69), respectively (Additional file 1: Table S6).

### Subgroup analyses to explore potential effect modifiers of GLP-1RA-associated chronic renal outcomes

**Fig. 3:**
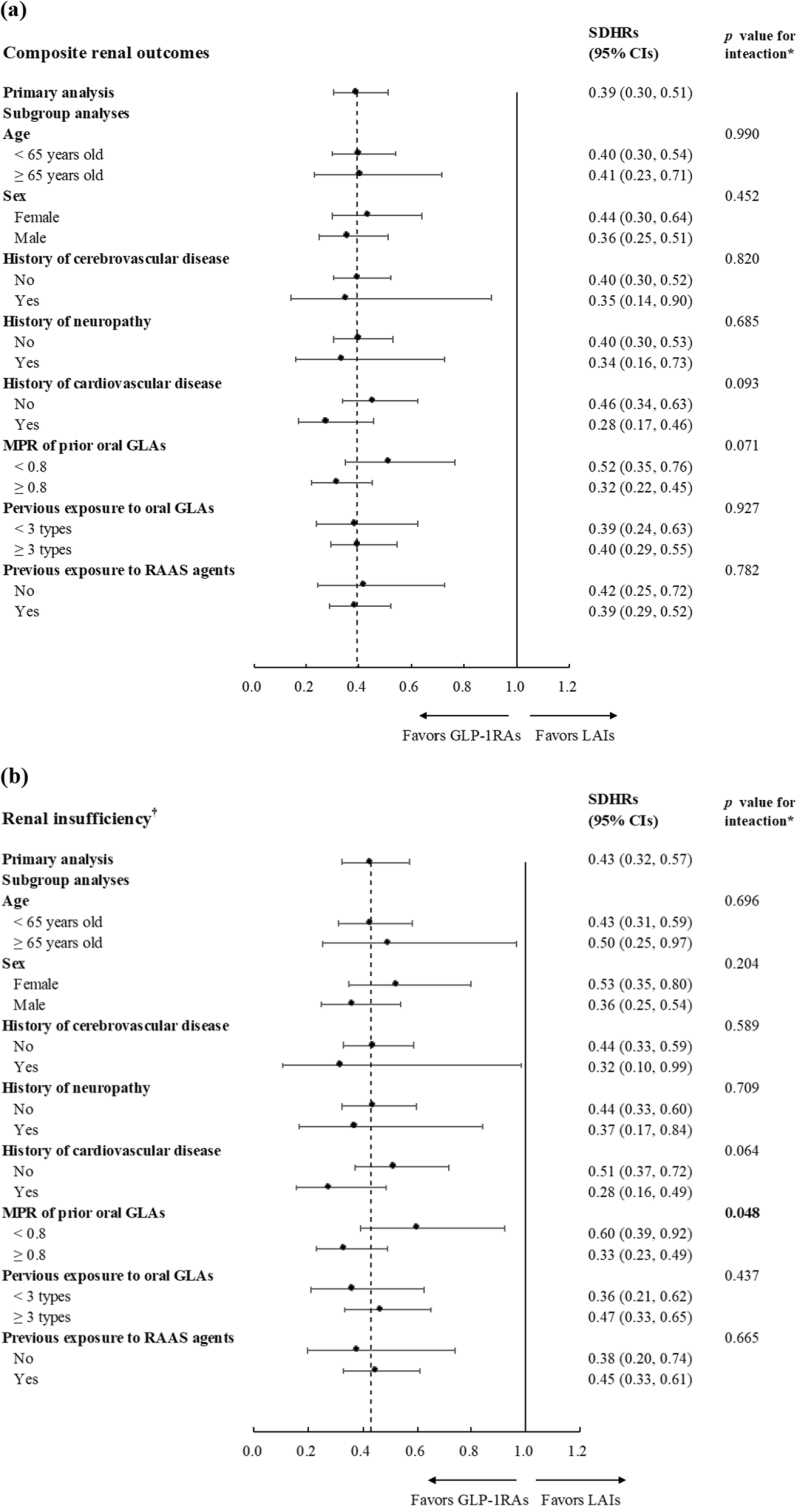

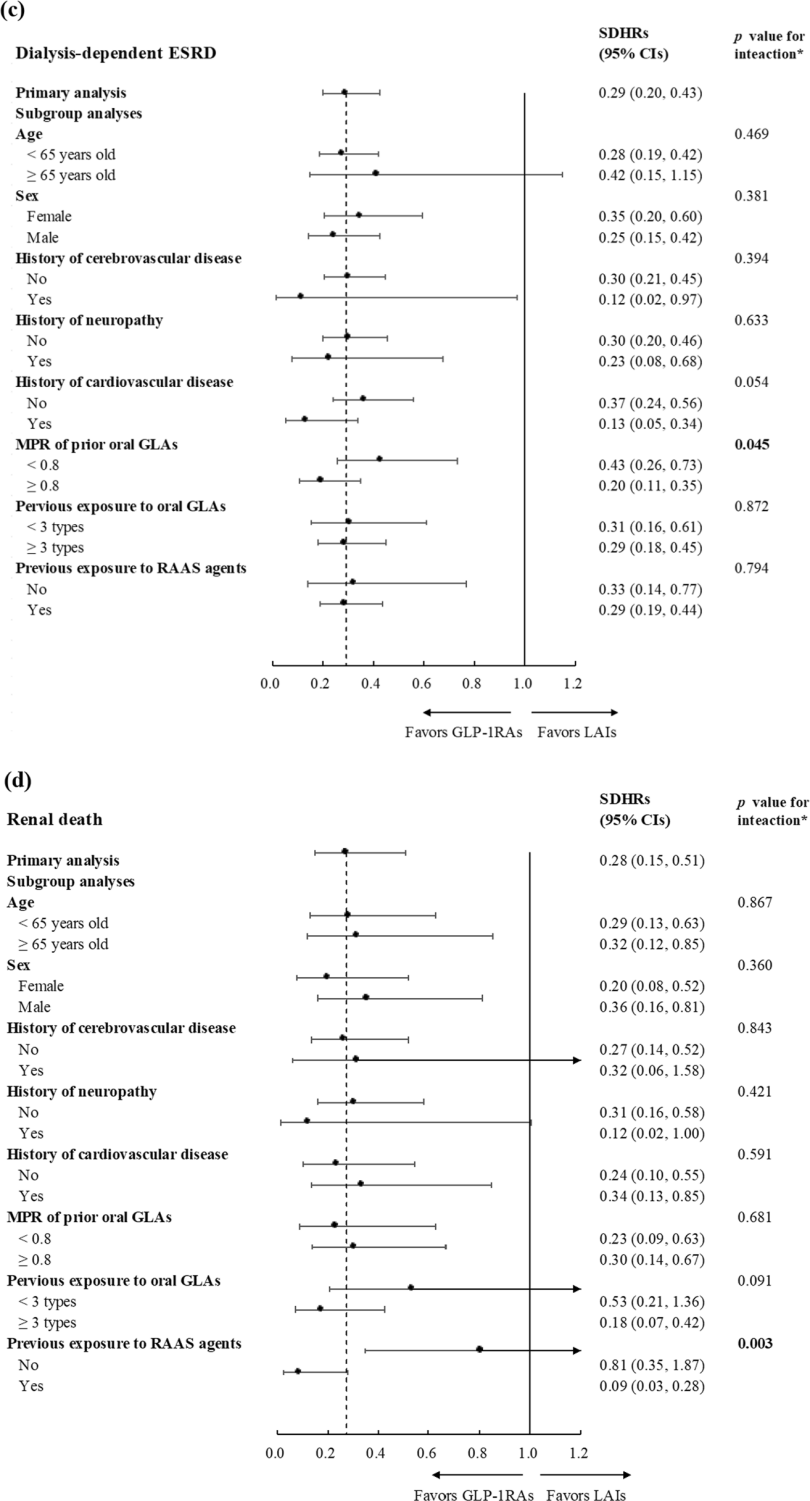
Forest plot for subgroup analysis results on (**a**) composite renal outcomes, (**b**) renal insufficiency (i.e., eGFR < 15 mL/min/1.73 m^2^), (**c**) dialysis-dependent end-stage renal disease, and (**d**) renal death. *eGFR* estimated glomerular filtration rate, *ESRD* end-stage renal disease, *SDHRs* subdistribution hazard ratios, *MPR* medication possession ratio, *GLAs* glucose-lowering agents, *RAAS* renin-angiotensin aldosterone system, *GLP-1RAs* glucagon-like peptide-1 receptor agonists, *LAIs* long-acting insulins. ^*^Bold* p* values for interaction tests indicate that treatment effect of GLP-1RAs versus LAIs on study outcome was significantly modified by given baseline characteristics. ^†^Renal insufficiency referred to eGFR < 15 mL/min/1.73 m^2^ and was determined by the stable use of erythropoiesis stimulating agents (ESAs) (i.e., at least two prescriptions of darbepoetin alfa or methoxy polyethylene glycol-epoetin beta, or four prescriptions of erythropoietin within three months), given that the reimbursement policy of Taiwan’s National Health Insurance program restricts the use of ESAs only to patients with stage 5 chronic kidney disease. This operational definition was also confirmed with clinical nephrologists

Overall, the use of GLP-1RAs versus LAIs led to a reduced risk of renal outcomes across different subgroups, except those aged ≥ 65 years old for dialysis-dependent ESRD and four subgroups for renal death (Fig. 3). Except for renal death, marginally significant effects of the interactions between CVD history (i.e., with versus without established CVDs) and treatment status (i.e., GLP-1RAs versus LAIs) on the risk of the composite chronic renal outcome (*p* = 0.093), renal insufficiency (i.e., eGFR < 15 mL/min/1.73 m^2^) (*p* = 0.064), and dialysis-dependent ESRD (*p* = 0.054) were found. Furthermore, marginally significant or significant effects of the interactions between the use of prior oral GLAs (i.e., MPR ≥ 0.8 versus < 0.8) and treatment status (i.e., GLP-1RAs versus LAIs) on the risk of the composite chronic renal outcome (*p* = 0.071), renal insufficiency (*p* = 0.048), and dialysis-dependent ESRD (*p* = 0.045) were found.

## Discussion

To our best knowledge, this study was the first to determine the progressive chronic renal outcomes of human-based GLP-1RA therapy (i.e., liraglutide and dulaglutide) among T2D patients who required intensive glycemic control and were potentially at risk of poor kidney functions. Our findings show that among T2D patients with poor glycemic control and initiating injectable GLAs, the use of GLP-1RAs versus LAIs was associated with lower risks of chronic renal disease progression (i.e., renal insufficiency as defined by eGFR < 15 mL/min/1.73 m^2^, dialysis-dependent ESRD, or renal death). A series of sensitivity analyses not only corroborated the primary analysis findings but also revealed the possibility of heterogenous treatment effects following GLP-1RA versus LAI therapies by patients’ clinical characteristics (e.g., disease history, medication adherence). Hence, this study adds supporting evidence to optimize clinical treatment decisions and promote precision medicine for averting substantial renal deterioration among clinically vulnerable T2D patients with poor glycemic control and at high risk of poor kidney prognosis.

### Existing evidence regarding GLP-1RA versus oral GLA therapies for chronic renal outcome

Considering the absence of trial results regarding the long-term renal effect of GLP-1RA therapy, real-world studies with the aim to assess the chronic renal outcomes of GLP-1RA versus oral GLA use have been conducted; however, the findings depend on the comparator drugs [11–13]. The HRs of GLP-1RAs versus dipeptidyl peptidase-4 inhibitors (DPP-4is) were in the range of 0.72 (95% CI: 0.53–0.98) [13] to 0.76 (0.68–0.85) [11] for the composite renal outcome, 0.73 (0.62–0.87) for renal dialysis or transplantation [11], and 0.72 (0.48–1.10) for renal death [11]. In contrast, those of sodium-glucose cotransporter-2 inhibitors versus GLP-1RAs were 0.77 (0.62–0.96) for the composite renal outcome and 0.53 (0.33–0.86) for ESRD [12]. Nevertheless, the findings obtained from comparative studies of GLP-1RAs and oral GLAs might not be generalizable to patients requiring injectable GLAs, who are likely to have poor glycemic control and are potentially at high risk of poor kidney outcomes compared to oral GLA users. Hence, an evaluation of the renal effects of GLA-1RA therapy among patients who require intensive glycemic control with injectable GLAs is urgently needed.

### Comparative effectiveness of GLP-1RA versus LAI therapies for chronic renal outcome

Among trials investigating the short-term renal benefits of GLP-1RA therapies [8–10, 26, 27], only the SURPASS-4 [27] and AWARD-7 [26] trials employed an active comparator design. They showed that the use of tirzepatide [27], a dual glucose-dependent insulinotropic polypeptide and GLP-1RA, or dulaglutide [26] versus insulin glargine had a comparable glucose-lowering effect but a better eGFR at 52 weeks of follow-up [26, 27]. In particular, the use of tirzepatide versus insulin glargine had a significantly lower risk on composite kidney endpoint which comprised time to first occurrence of eGFR decline of at least 40% from baseline, ESRD, death owing to kidney failure, or new-onset macroalbuminuria (HR: 0.58; 95% CI: 0.43–0.80) [27]. However, such renal benefits may not be generalized to patients only using GLP1-RA therapy. Furthermore, comparative kidney outcomes associated with exenatide versus LAI use have been examined in a previous study [28], which however may not be generalizable to patients using human-based GLP-1RAs (i.e., liraglutide and dulaglutide). Recently, the FLOW trial, an ongoing trial, prospectively evaluates the effect of semaglutide versus insulin glargine on renal outcomes (including time to first kidney failure, persistent ≥ 50% reduction in eGFR or death from kidney or CV causes) in patients with T2D and CKD [29]. This trial is expected to complete in late 2024.

To our best knowledge, the present study is the first to assess the comparative effectiveness of GLP-1RAs (i.e., liraglutide and dulaglutide) versus LAIs for a series of progressive chronic renal outcomes. The results of primary and sensitivity analyses suggest that in a mean 2.64-year follow-up, the use of GLP-1RAs versus LAIs significantly decreased the risk of the composite chronic renal outcome by 51–63%, renal insufficiency (i.e., eGFR < 15 mL/min/1.73 m^2^) by 50–63%, dialysis-dependent ESRD by 54–71%, and renal death by 47–72% (Fig. 2). Of note, injectable GLAs, including GLP-1RAs, are commonly initiated at a later treatment course among real-world T2D populations, especially for patients with persistent hyperglycemia despite the use of multiple oral GLAs [30, 31]. Our study patients had multiple oral GLA therapies prior to the initiation of GLP-1RAs or LAIs, which could be attributable to the reimbursement policy of Taiwan’s NHI program where the use of GLP-1RAs was restricted to T2D patients with glycated hemoglobin (HbA_1c_) greater than 8.5% for at least 6 months. Previous real-world studies in Taiwan showed that the HbA_1c_ level of patients at the beginning of GLP-1RAs generally ranged between 9.2 and 9.4% [32–34]. Such high HbA_1c_ levels (i.e., 9% and above) have been correlated with undesired kidney outcomes, including ESRD [35, 36], leading to high mortality, poor quality of life [37], and considerable healthcare spending [38]. Hence, given the remarkable renal outcomes of GLA-1RA therapies shown in this study, timely initiation of GLP-1RAs among T2D patients who require intensive glycemic control with injectable GLAs is suggested to avert the progression to renal failure/dialysis or death, leading to better patient quality of life and better economic outcomes.

### Effect modifiers for GLP-1RA-associated kidney outcomes

Our exploratory analyses identified CVD history and the MPR of prior oral GLAs as potential effect modifiers (Fig. 3). That is, CVD history had marginally significant modification effects on the GLP-1RA-associated composite renal outcome, renal insufficiency (i.e., eGFR < 15 mL/min/1.73 m^2^), and dialysis-dependent ESRD, while the MPR of prior oral GLAs had significant modification effects on the renal insufficiency and dialysis-dependent ESRD. Our findings may be explained by existing non-clinical and clinical studies. Specifically, CV impairment could worsen renal functions [39]. Owing to drug action on cardiomyocytes, GLP-1RAs could enhance nitric oxide production, glucose uptake, and coronary flow, thereby averting myocardial ischemia and ventricular dysfunction [40]. Such CV improvement could also benefit kidney systems. Together with our results, one may expect that compared to those without CVD history, patients with established CVDs will gain more kidney benefits (i.e., slowed or preserved renal function) from timely intervention with GLP-1RAs.

Non-adherence to GLAs is a well-known risk factor for treatment failure (i.e., uncontrolled HbA_1c_), and is associated with increased comorbidities and diabetes-related complications [41, 42]. We found that patients who previously achieved greater adherence to oral GLAs (i.e., MPR ≥ 0.8) had a lower risk of poor progressive chronic renal outcomes (i.e., renal insufficiency as defined by eGFR < 15 mL/min/1.73 m^2^, and dialysis-dependent ESRD; Fig. 3) compared to those with less adherence (i.e., MPR < 0.8). Therefore, greater glycemic control and renal benefits following GLP-1RA therapy among patients with good medication adherence are expected. In contrast, for those with poor adherence to prior oral GLAs, education or close monitoring to enhance medication adherence is important for optimizing the treatment effects of GLP-1RAs.

### Study limitations

Several limitations of this study should be acknowledged. First, due to the lack of laboratory (e.g., HbA1c, blood pressure, and albuminuria) and behavioral data in claims-based data, residual unmeasured confounding might exist in our study. Several methodology efforts therefore were carried out to minimize the possibility of unmeasured confounding effect to study results, including (1) the adjustment of well-known proxies (e.g., prior use of GLAs for the level of glycemic control, past history of diabetic nephropathy, neuropathy and retinopathy for the severity of diabetes, prior exposure of anti-hypertensive medications for the status of blood pressure control) in the PS estimation in primary analysis, (2) using the hdPS and considering baseline patient characteristics from empirical data in the NHIRD in a sensitivity analysis, and 3) applying 3P-MACE as positive control outcomes to support the validity of our study procedures. Of noted, our finding in positive control outcome analysis (i.e., the magnitude of CV effect: 29% of reduction in 3P-MACE risk associated with GLP-1RA versus LAI use) falls in the range of 19–41% of risk reduction shown in previous studies [4, 25], suggesting that the issue of unmeasured confounding effect in this study might be negligible. Second, the treatment pattern (e.g., adherence) in real-world patient populations may be suboptimal and could consequently affect treatment outcomes. Therefore, in additional to the ITT scenario in the primary analyses, AST scenario analyses were performed with consideration of the discontinuation or switching of study drugs in this real-world study. Third, the primary analyses might be limited to PSM-derived GLP-1RA and LAI users with similar baseline characteristics because these patients might not be representative of drug users with diverse clinical characteristics in real-world settings. Therefore, sensitivity analyses using various PS weighting techniques (i.e., IPTW, stabilized PTW, and SMRW) were further performed, where the original users of study drugs were retained to ensure the diversity of study cohorts to reflect real-world patients. Fourth, the subgroup analyses stratified by baseline patient characteristics are considered as exploratory in nature. Hence, future research is warranted to corroborate our findings. Lastly, the generalizability of study results on renal insufficiency might be limited. Since the stable use of ESAs (i.e., only for patients with eGFR level < 15 ml/min/1.73m^2^ under Taiwan’s NHI reimbursement policy) was applied as a surrogate indicator for renal insufficiency [18], our study patients having renal insufficiency were likely to be severe cases.

## Conclusions

Compared to LAIs, using GLP-1RAs was associated with lower risk of progressive chronic renal outcomes among a real-world T2D patient population that required intensive GLA therapy and was at risk of poor renal progression. GLP-1RA therapy for these vulnerable patients should thus be timely administered. Prioritization of GLP-1RAs for the patients with established CVDs or optimal adherence to prior oral GLAs should be considered to maximize the renal benefits of using GLP-1RA therapy.

### Supplementary Information

**Additional file 1: Table S1.** Operational definitions of study outcomes. **Table S2.** Proportions of individual GLP-1RAs and LAIs before and after propensity-score matching. **Table S3.** Baseline characteristics of study cohorts before and after propensity score matching. **Table S4.** Event rates and hazard ratios of renal outcomes associated with use of GLP-1RAs versus LAIs (as-treated scenario analyses). **Table S5.** Sensitivity analysis results of renal outcomes associated with use of GLP-1RAs versus LAIs. **Table S6.** Event rates and hazard ratios of cardiovascular outcomes associated with use of GLP-1RAs versus LAIs (positive control outcome analyses). **Figure S1.** Flow chart of cohort selection. **Figure S2.** Kernel density plots of propensity score distributions for study cohorts (a) before and (b) after matching.

## Data Availability

Raw data were generated at Taiwan’s National Health Insurance Research Database. Derived data supporting the findings of this study are available from the corresponding author Dr. Huang-Tz Ou on request.

## Abbreviations

3P-MACE: Three-point major adverse cardiovascular event
AST: As-treated
CV: Cardiovascular
CVDs: Cardiovascular diseases
CKD: Chronic kidney disease
DPP-4is: Dipeptidyl peptidase-4 inhibitors
eGFR: Estimated Glomerular filtration rate
ESAs: Erythropoiesis stimulating agents
ESRD: End-stage renal disease
GLAs: Glucose-lowering agents
GLP-1RAs: Glucagon-like peptide-1 receptor agonists
hdPS: High-dimensional propensity score
HbA_1c_: Glycated hemoglobin
IPTW: Inverse probability of treatment weighting
ITT: Intention-to-treat
LAIs: Long-acting insulins
MPR: Medication possession ratio
NHIRD: National Health Insurance Research Database
NHI: National Health Insurance
PSM: Propensity score matching
SMD: Standardized mean difference
SDHR: Subdistribution hazard ratio
SMRW: Standardized mortality ratio weighting
T2D: Type 2 diabetes
