# Supplementary material for: Chronic kidney outcomes associated with GLP-1 receptor agonists versus long-acting insulins among type 2 diabetes patients requiring intensive glycemic control: a nationwide cohort study
Source: Cardiovasc Diabetol. 2023 Oct 4;22:272. doi: 10.1186/s12933-023-01991-5 (PMC10552437; doi:10.1186/s12933-023-01991-5)
Supplement: Supplementary file 1 — Additional file 1: Table S1. Operational definitions of study outcomes. Table S2. Proportions of individual GLP-1RAs and LAIs before and after propensity-score matching. Table S3. Baseline characteristics of study cohorts before and after propensity score matching. Table S4. Event rates and hazard ratios of renal outcomes associated with use of GLP-1RAs versus LAIs (as-treated scenario analyses). Table S5. Sensitivity analysis results of renal outcomes associated with use of GLP-1RAs versus LAIs. Table S6. Event rates and hazard ratios of cardiovascular outcomes associated with use of GLP-1RAs versus LAIs (positive control outcome analyses). Figure S1. Flow chart of cohort selection. Figure S2. Kernel density plots of propensity score distributions for study cohorts (a) before and (b) after matching. [file 12933_2023_1991_MOESM1_ESM.docx]

Table S1. Operational definitions of study outcomes

Table S2. Proportions of individual GLP-1RAs and LAIs before and after propensity-score matching

Table S3. Baseline characteristics of study cohorts before and after propensity score matching

Table S4. Event rates and hazard ratios of renal outcomes associated with use of GLP-1RAs versus LAIs (as-treated scenario analyses)

Table S5. Sensitivity analysis results of renal outcomes associated with use of GLP-1RAs versus LAIs

Table S6. Event rates and hazard ratios of cardiovascular outcomes associated with use of GLP-1RAs versus LAIs (positive control outcome analyses)

Figure S1. Flow chart of cohort selection

Figure S2. Kernel density plots of propensity score distributions for study cohorts (a) before and (b) after matching

Table S1. Operational definitions of study outcomes

| **Outcomes** | **Operational definitions** | | **Data files in NHIRD** |
| --- | --- | --- | --- |
| *Outcome defined by ATC codes* | | | |
| Renal insufficiency defined by the stable use of ESAs (i.e., estimated glomerular filtration rate < 15 mL/min/1.73 m^2^)^*^ | B03XA01, B03XA02, B03XA03 | | Outpatient, inpatient, and emergency departments |
| *Outcomes defined by ICD-9/10-CM diagnosis codes* | | | |
|  | ICD-9-CM disease codes | ICD-10-CM disease codes |  |
| Dialysis-dependent ESRD | 585 | N185, N186 | Registry for Catastrophic Illness Patients |
| Renal death | 580-589 | N00-N07, N17-N19, N25- N27 | Cause of Death Data |
| Myocardial infarction | 410 | I21 | Inpatient and emergency departments |
| Stroke | 430, 431-434 | I60-I63 | Inpatient and emergency departments |
| Cardiovascular death | 390-392,393-398,410-414,420-429 | I01-I02.0, I05-I09, I20-I25, I27, I30-I52 | Cause of Death Data |

Abbreviations: NHIRD, National Health Insurance Research Database; ATC, Anatomical Therapeutic Chemical; ESAs, erythropoiesis stimulating agents; GFR, estimated glomerular filtration rate; ICD-9/10-CM, International Classification of Diseases-ninth/tenth versions Clinical Modification.

^*^Renal insufficiency referred to eGFR < 15 mL/min/1.73 m^2^ and was determined by the stable use of ESAs (i.e., at least two prescriptions of darbepoetin alfa or methoxy polyethylene glycol-epoetin beta, or four prescriptions of erythropoietin within three months), given that the reimbursement policy of Taiwan’s National Health Insurance program restricts the use of ESAs only to patients with stage 5 chronic kidney disease. This operational definition was also confirmed with clinical nephrologists.

Table S2. Proportions of individual GLP-1RAs and LAIs before and after propensity-score matching

|  | LAIs (%) | | GLP-1RAs (%) | |
| --- | --- | --- | --- | --- |
|  | Insulin glargine | Insulin determir | Liraglutide | dulaglutide |
| Before PSM | 68.2 | 31.8 | 53.8 | 46.2 |
| After PSM | 69.9 | 30.1 | 46.5 | 53.5 |

Abbreviations: LAIs, long-acting insulins; GLP-1RAs, glucagon-like peptide-1 receptor agonists; PSM, propensity-score matching.

Table S3. Baseline characteristic of study cohorts before and after propensity score matching

| Characteristics | Before PSM | |  | After PSM | |  |
| --- | --- | --- | --- | --- | --- | --- |
|  | LAIs | GLP-1RAs | SMD^*^ | LAIs | GLP-1RAs | SMD^*^ |
| Number of subjects | 49,570 | 7,643 |  | 7,279 | 7,279 |  |
| **Demographics at index date**^†^ |  |  |  |  |  |  |
| Age (years, mean ± SD) | 56.2 ± 14.7 | 48.7 ± 12.5 | -0.55 | 49.2 ± 13.4 | 49.1 ± 12.4 | 0.00 |
| Female (%) | 40.3 | 50.2 | 0.46 | 49.1 | 49.5 | 0.01 |
| Year of index date^†^ (%) |  |  |  |  |  |  |
| 2013 | 10.9 | 3.0 | -0.31 | 2.7 | 3.1 | 0.03 |
| 2014 | 13.1 | 4.7 | -0.30 | 4.6 | 4.9 | 0.01 |
| 2015 | 15.2 | 9.4 | -0.18 | 9.7 | 9.7 | 0.00 |
| 2016 | 16.8 | 14.1 | -0.08 | 14.9 | 14.3 | -0.02 |
| 2017 | 20.1 | 27.8 | 0.18 | 27.8 | 27.6 | 0.00 |
| 2018 | 23.9 | 41.1 | 0.38 | 40.4 | 40.4 | 0.00 |
| **Surrogate indicators for diabetes severity** |  |  |  |  |  |  |
| Number of oral GLAs prescribed in year before index date^†^ (mean ± SD) | 2.3 ± 1.5 | 2.7 ± 1.2 | -0.30 | 2.7 ± 1.2 | 2.7 ± 1.2 | -0.04 |
| Recent use of prandial insulins in month before index date^†^ (%) |  |  |  |  |  |  |
| Rapid-acting insulins | 0.4 | 0.3 | -0.03 | 0.3 | 0.3 | -0.01 |
| Intermediate-acting insulins | 0.6 | 0.2 | -0.07 | 0.1 | 0.2 | 0.01 |
| Mixed insulins | 3.8 | 3.8 | 0.00 | 4.1 | 3.8 | -0.01 |
| **Diabetes-related complications and comorbidities in year before index date**^†^ **(%)** |  |  |  |  |  |  |
| Nephropathy | 25.0 | 24.3 | -0.02 | 25.6 | 24.6 | -0.02 |
| Neuropathy | 9.7 | 7.6 | -0.08 | 8.1 | 7.8 | -0.01 |
| Retinopathy | 11.9 | 11.0 | -0.03 | 11.3 | 11.2 | 0.00 |
| Proliferative diabetic retinopathy | 3.1 | 2.1 | -0.07 | 2.2 | 2.1 | 0.00 |
| Non-proliferative diabetic retinopathy | 5.0 | 4.6 | -0.02 | 4.8 | 4.7 | -0.01 |
| Diabetic macular edema | 2.7 | 2.4 | -0.02 | 2.6 | 2.5 | -0.01 |
| Peripheral vascular disease | 4.8 | 2.8 | -0.11 | 3.0 | 2.8 | -0.01 |
| Hemorrhage stroke | 1.8 | 0.5 | -0.12 | 0.4 | 0.5 | 0.01 |
| Ischemic stroke | 6.9 | 2.4 | -0.21 | 2.4 | 2.5 | 0.01 |
| Transient ischemic attack | 1.7 | 1.0 | -0.05 | 1.3 | 1.1 | -0.02 |
| Cardiovascular disease | 25.1 | 18.7 | -0.15 | 19.1 | 19.0 | 0.00 |
| Heart failure | 5.3 | 3.3 | -0.10 | 3.4 | 3.4 | 0.00 |
| Ischemic heart disease | 13.3 | 12.1 | -0.04 | 12.5 | 12.2 | -0.01 |
| Diabetic ketoacidosis | 18.5 | 26.9 | 0.20 | 25.6 | 26.1 | 0.01 |
| Hyperglycemic hyperosmolar state | 19.1 | 26.5 | 0.18 | 24.9 | 25.6 | 0.02 |
| Hypoglycemia | 1.2 | 0.6 | -0.07 | 0.6 | 0.6 | 0.00 |
| Obesity | 1.4 | 9.1 | 0.35 | 5.9 | 6.5 | 0.03 |
| Hypertension | 52.6 | 54.4 | 0.04 | 55.9 | 54.7 | -0.02 |
| **Previous GLA exposure in year before index date**^†^ **(%)** |  |  |  |  |  |  |
| Metformin | 69.8 | 90.1 | 0.52 | 90.4 | 89.7 | -0.02 |
| Sulfonylureas | 61.4 | 64.1 | 0.06 | 67.6 | 65.6 | -0.04 |
| Meglitinides | 10.2 | 7.5 | -0.1 | 8.0 | 7.6 | -0.01 |
| Thiazolidinediones | 15.7 | 20.6 | 0.13 | 20.9 | 20.7 | 0.00 |
| Acarbose | 19.2 | 18.5 | -0.02 | 18.6 | 18.9 | 0.01 |
| DPP4is | 49.5 | 65.1 | 0.32 | 67.1 | 65.3 | -0.04 |
| SGLT2is | 0.2 | 0.5 | 0.06 | 0.5 | 0.5 | -0.01 |
| **Medication possession ratio of prior GLA use in year before index date**^†^ **(%)** | 0.5 (0.4) | 0.7 (0.3) | 0.59 | 0.7 (0.3) | 0.7 (0.3) | -0.02 |
| **Cardiovascular and kidney diseases-related medication history in year before index date**^†^ **(%)** |  |  |  |  |  |  |
| Lipid-lowering agents | 52.1 | 74.5 | 0.48 | 74.7 | 74.1 | -0.02 |
| Alpha blockers | 4.0 | 3.3 | -0.04 | 3.6 | 3.3 | -0.02 |
| Beta blockers | 25.8 | 27.9 | 0.05 | 28.3 | 28.0 | -0.01 |
| RAAS agents | 43.7 | 54.2 | 0.21 | 54.7 | 54.1 | -0.01 |
| Diuretics | 17.6 | 12.8 | -0.13 | 13.5 | 12.8 | -0.02 |
| Calcium channel blockers | 27.0 | 22.6 | -0.10 | 23.3 | 22.8 | -0.01 |
| Anti-arrhythmics | 1.9 | 1.4 | -0.04 | 1.4 | 1.4 | 0.00 |
| Cardiac glycosides | 1.9 | 0.8 | -0.10 | 0.8 | 0.8 | 0.00 |
| Vasodilators | 22.3 | 18.2 | -0.10 | 18.9 | 18.5 | -0.01 |
| Antiplatelets | 26.5 | 22.1 | -0.10 | 22.8 | 22.6 | 0.00 |
| Anticoagulants | 2.8 | 1.6 | -0.08 | 1.7 | 1.7 | 0.00 |

Abbreviations: PSM, propensity-score matching; LAI, long-acting insulin; GLP-1RA, glucagon-like peptide-1 receptor agonist; SMD, standard mean difference; SD, standard deviation; GLA, glucose-lowering agent; DPP4is, dipeptidyl peptidase-4 inhibitors; SGLT2is, sodium-glucose cotransporter-2 inhibitors; RAAS, renin-angiotensin aldosterone system.

Notes:

^*^SMD value greater than 0.1 suggests statistically significant between-group difference in baseline characteristics.

^†^Index date was defined as date of initiation of GLP1RA or LAI therapy.

Table S4. Event rates and hazard ratios of renal outcomes associated with use of GLP-1RAs versus LAIs (as-treated scenario analyses)

|  | GLP-1RAs (n=7,279) | | LAIs (n=7,279) | | SDHR (95% CI) of GLP-1RAs versus LAIs |
| --- | --- | --- | --- | --- | --- |
|  | Number of events | Event rate (events/100 pys) | Number of events | Event rate (events/100 pys) |  |
| Composite renal outcome^*^ | 35 | 0.27 | 100 | 0.74 | 0.36 (0.25-0.53) |
| Renal insufficiency (i.e., eGFR < 15 mL/min/1.73 m^2^)^†^ | 34 | 0.26 | 87 | 0.65 | 0.40 (0.27-0.60) |
| Dialysis-dependent ESRD | 11 | 0.08 | 57 | 0.42 | 0.20 (0.11-0.38) |
| Renal death | 3 | 0.02 | 7 | 0.05 | 0.47 (0.11-1.68) |

Abbreviations: GLP-1RAs, glucagon-like peptide-1 receptor agonists; LAIs, long-acting insulins; SDHR, subdistribution hazard ratio; pys, person-years; eGFR, estimated glomerular filtration rate; ESRD, end-stage renal disease.

^*^Composite renal outcome includes stable use of ESA, dialysis-dependent ESRD, and renal death.

^†^Renal insufficiency referred to eGFR < 15 mL/min/1.73 m^2^ and was determined by the stable use of erythropoiesis stimulating agents (ESAs) (i.e., at least two prescriptions of darbepoetin alfa or methoxy polyethylene glycol-epoetin beta, or four prescriptions of erythropoietin within three months), given that the reimbursement policy of Taiwan’s National Health Insurance program restricts the use of ESAs only to patients with stage 5 chronic kidney disease. This operational definition was also confirmed with clinical nephrologists.

Table S5. Sensitivity analysis results of renal outcomes associated with use of GLP-1RAs versus LAIs

|  | SDHR (95% CI) associated with GLP1RA versus LAI use | | | | |
| --- | --- | --- | --- | --- | --- |
|  | Primary analysis | Sensitivity analysis | | | |
|  | PSM cohort | IPTW cohort^*^ | Stabilized IPTW cohort^*^ | SMRW cohort^*^ | hdPS-matched cohort |
| Composite renal outcome^†^ | 0.39 (0.30-0.51) | 0.37 (0.34, 0.39) | 0.37 (0.30, 0.44) | 0.39 (0.32, 0.48) | 0.42 (0.30, 0.58) |
| Renal insufficiency (i.e., eGFR < 15 mL/min/1.73 m^2^)^‡^ | 0.43 (0.32-0.57) | 0.37 (0.34, 0.40) | 0.37 (0.31, 0.44) | 0.39 (0.32, 0.48) | 0.44 (0.30, 0.63) |
| Dialysis-dependent ESRD | 0.29 (0.20-0.43) | 0.31 (0.29, 0.34) | 0.32 (0.26, 0.39) | 0.34 (0.27, 0.43) | 0.25 (0.15, 0.41) |
| Renal death | 0.28 (0.15-0.51) | 0.31 (0.28, 0.34) | 0.31 (0.24, 0.40) | 0.34 (0.26, 0.44) | 0.40 (0.19, 0.83) |

Abbreviations: SDHR, subdistribution hazard ratio; GLP-1RAs, glucagon-like peptide-1 receptor agonists; LAIs, long-acting insulins; PSM, propensity-score matched; IPTW, inverse probability of treatment weighting; SMRW, Standardized mortality ratio weighting; hdPS, high-dimensional propensity score; eGFR, estimated glomerular filtration rate; ESRD, end-stage renal disease.

Notes:

^*^We removed patients whose propensity scores were either more than 0.95 or less than 0.05; the weights were then estimated based on the trimmed populations. Weights in the IPTW approach were estimated as follows: Weight_GLP1RAs_=1/PS and Weight_LAIs_=1/(1-PS). Weights in the stabilized IPTW cohort were estimated as follows: Weight_GLP1RAs_=Prevalence of GLP1RA users (%)/PS and Weight_LAIs_= Prevalence of LAI users (%)/(1-PS). Weights in the SMRW approach were estimated as follows: Weight_GLP1RAs_=1 and Weight_LAIs_=PS/(1-PS).

^†^Composite renal outcome includes stable use of ESA, dialysis-dependent ESRD, and renal death.

^‡^Renal insufficiency referred to eGFR < 15 mL/min/1.73 m^2^ and was determined by the stable use of erythropoiesis stimulating agents (ESAs) (i.e., at least two prescriptions of darbepoetin alfa or methoxy polyethylene glycol-epoetin beta, or four prescriptions of erythropoietin within three months), given that the reimbursement policy of Taiwan’s National Health Insurance program restricts the use of ESAs only to patients with stage 5 chronic kidney disease. This operational definition was also confirmed with clinical nephrologists.

Table S6. Event rates and hazard ratios of cardiovascular outcomes associated with use of GLP-1RAs versus LAIs (positive control outcome analyses)

|  | GLP-1RAs | | LAIs | | SDHR (95% CI) of GLP-1RAs versus LAIs |
| --- | --- | --- | --- | --- | --- |
|  | Number of events | Event rate (events/100 pys) | Number of events | Event rate (events/100 pys) |  |
| 3P-MACE^*^ | 225 | 1.18 | 318 | 1.68 | 0.71 (0.60-0.84) |
| Non-fatal myocardial infarction | 72 | 0.37 | 99 | 0.52 | 0.73 (0.54-0.99) |
| Non-fatal stroke | 131 | 0.68 | 174 | 0.91 | 0.76 (0.60-0.95) |
| Cardiovascular death | 46 | 0.24 | 95 | 0.49 | 0.49 (0.34-0.69) |

Abbreviations: GLP-1RAs, glucagon-like peptide-1 receptor agonists; LAIs, long-acting insulins; SDHR, subdistribution hazard ratio; pys, person-years; 3P-MACE, three-point major adverse cardiovascular event.

^*^3P-MACE comprises non-fatal myocardial infarction, non-fatal stroke, and cardiovascular death.

Figure S1. Flow chart of cohort selection

| Patients diagnosed with type 2 diabetes and with stable use^*^ of GLP-1RAs or LAIs in period of 2013 to 2018 (n=99,889) |
| --- |

1. Age < 18 years old at index date^†^ or undefined sex (n=1,633)
2. Exposure to GLP-1RAs or LAIs in year prior to index date^†^ (n=23,378)
3. Combination use of GLP-1RAs and LAIs at index date^†^ (n=1,210)
4. Use of basal-bolus insulin regimens or premixed insulin at index date^†^ (n=12,593)
5. History of ESA use, ESRD, or renal replacement in year prior to index date^†^ (n=3,862)

| Incident new-users of GLP-1RAs (n=7,643) or LAIs (n=49,570) |
| --- |

| Propensity-score-matched pairs of GLP-1RAs and LAIs (n=7,279) |
| --- |

Abbreviations: GLP-1RAs, glucagon-like peptide-1 receptor agonists; LAIs, long-acting insulins; ESAs, erythropoiesis stimulating agents; ESRD, end-stage renal disease.

Notes:

^*^Stable use was defined as patients with at least three prescriptions of study drug (i.e., GLP-1RAs or LAIs) with any gaps between two consecutive drug refills of less than 30 days.

^†^Index date refers to date of initiation of GLP-1RA or LAI therapy.

Figure S2. Kernel density plots of propensity score (PS) distributions for study cohorts (a) before and (b) after matching

| (a) before matching | (b) after matching |
| --- | --- |
| 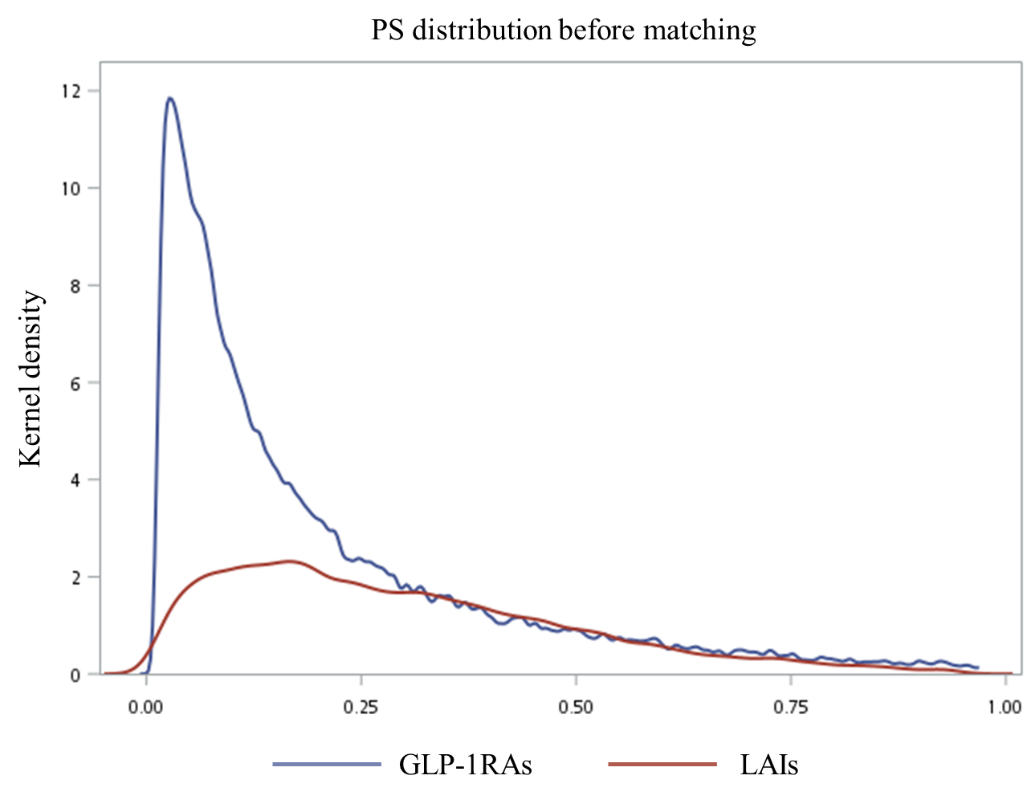 | 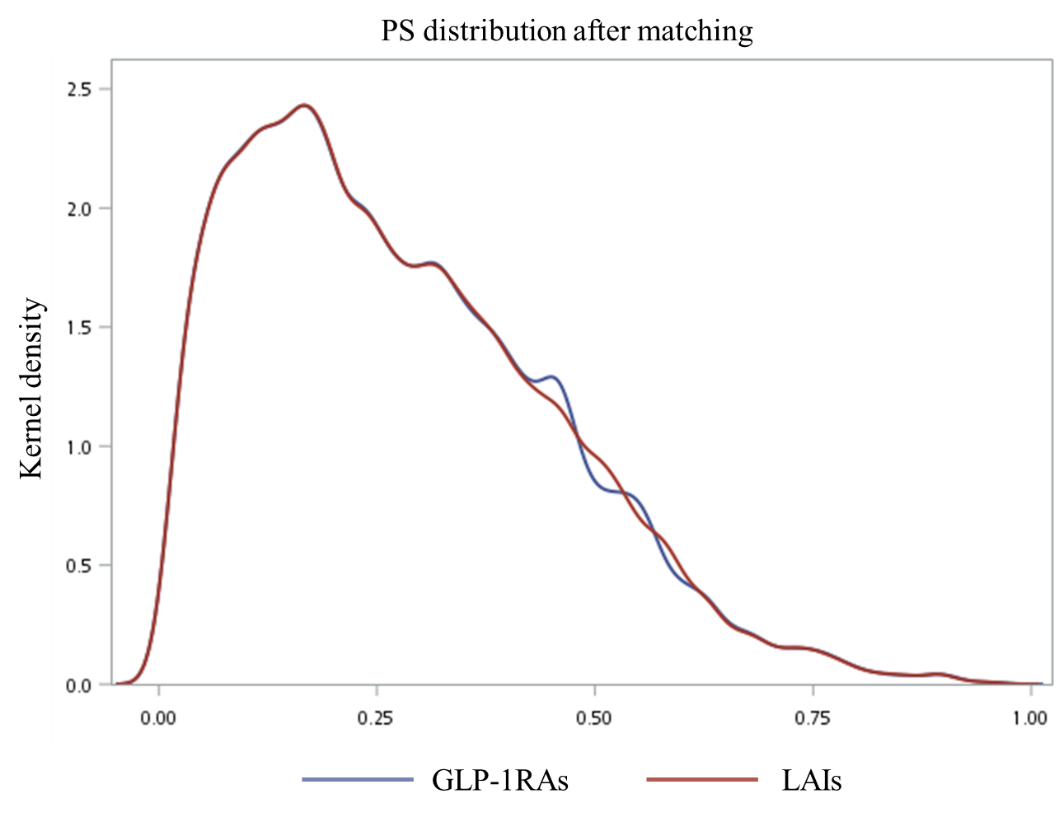 |

Abbreviations: GLP-1RAs, glucagon-like peptide-1 receptor agonists; LAIs, long-acting insulins.
